# Supplementary material for: Can good ESG performance of listed companies reduce abnormal stock price volatility? Mediation effects based on investor attention
Source: PLoS One. 2024 Sep 6;19(9):e0307535. doi: 10.1371/journal.pone.0307535 (PMC11379154; doi:10.1371/journal.pone.0307535)

**Table 1. Variable description and definition.**


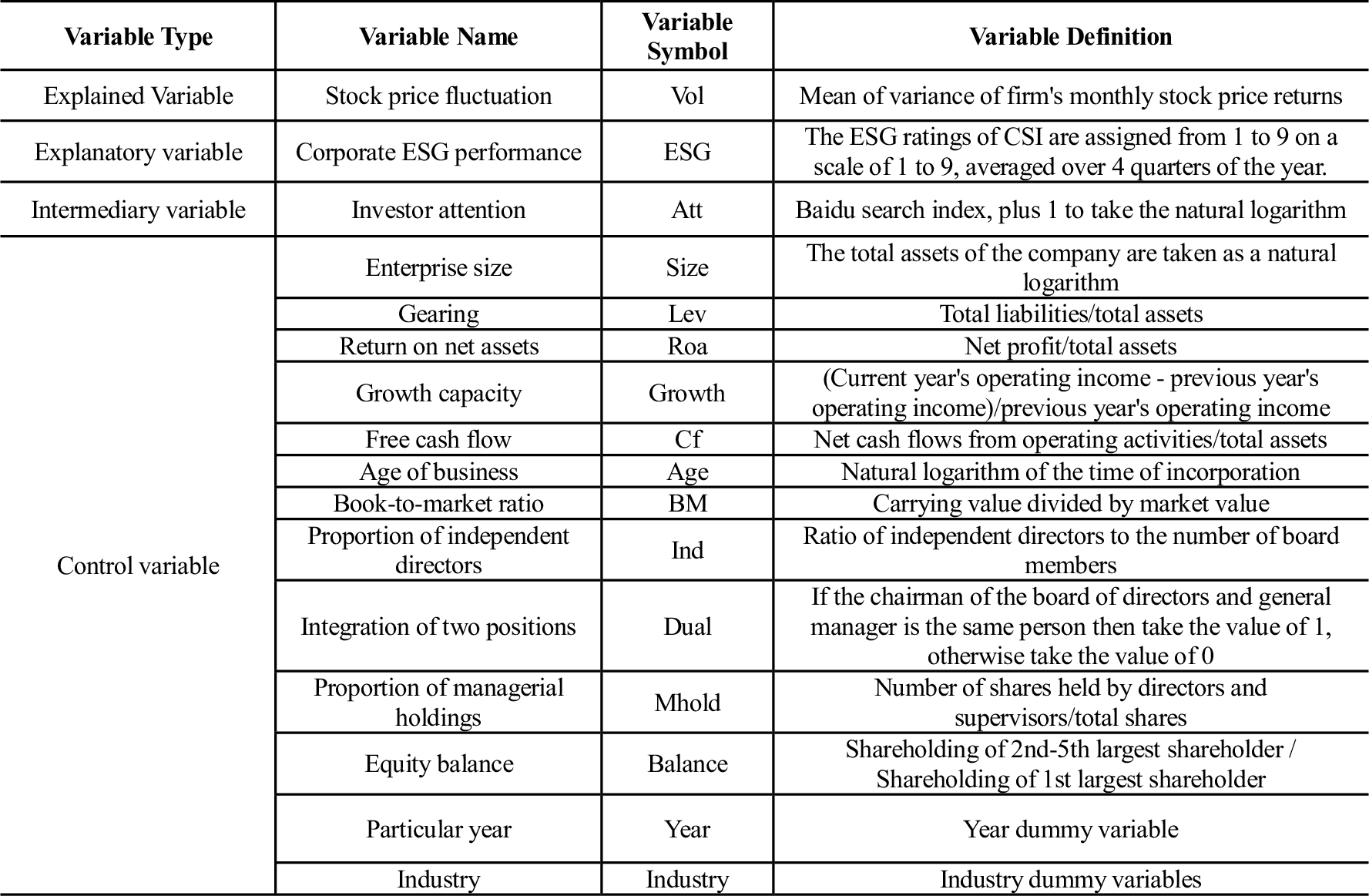


**Table 2. Descriptive statistics.**


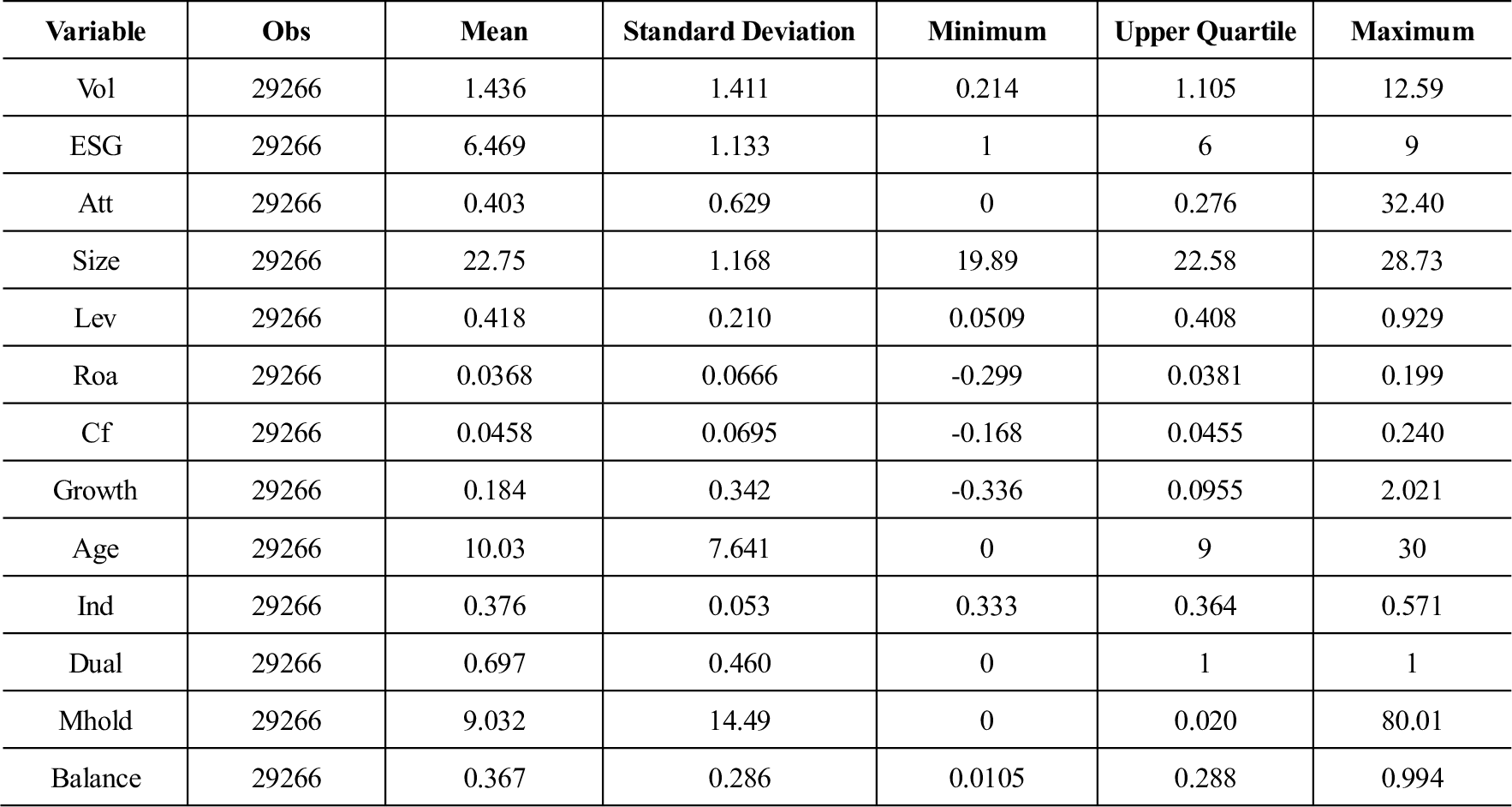


**Table 3. Correlation analysis results.**

**
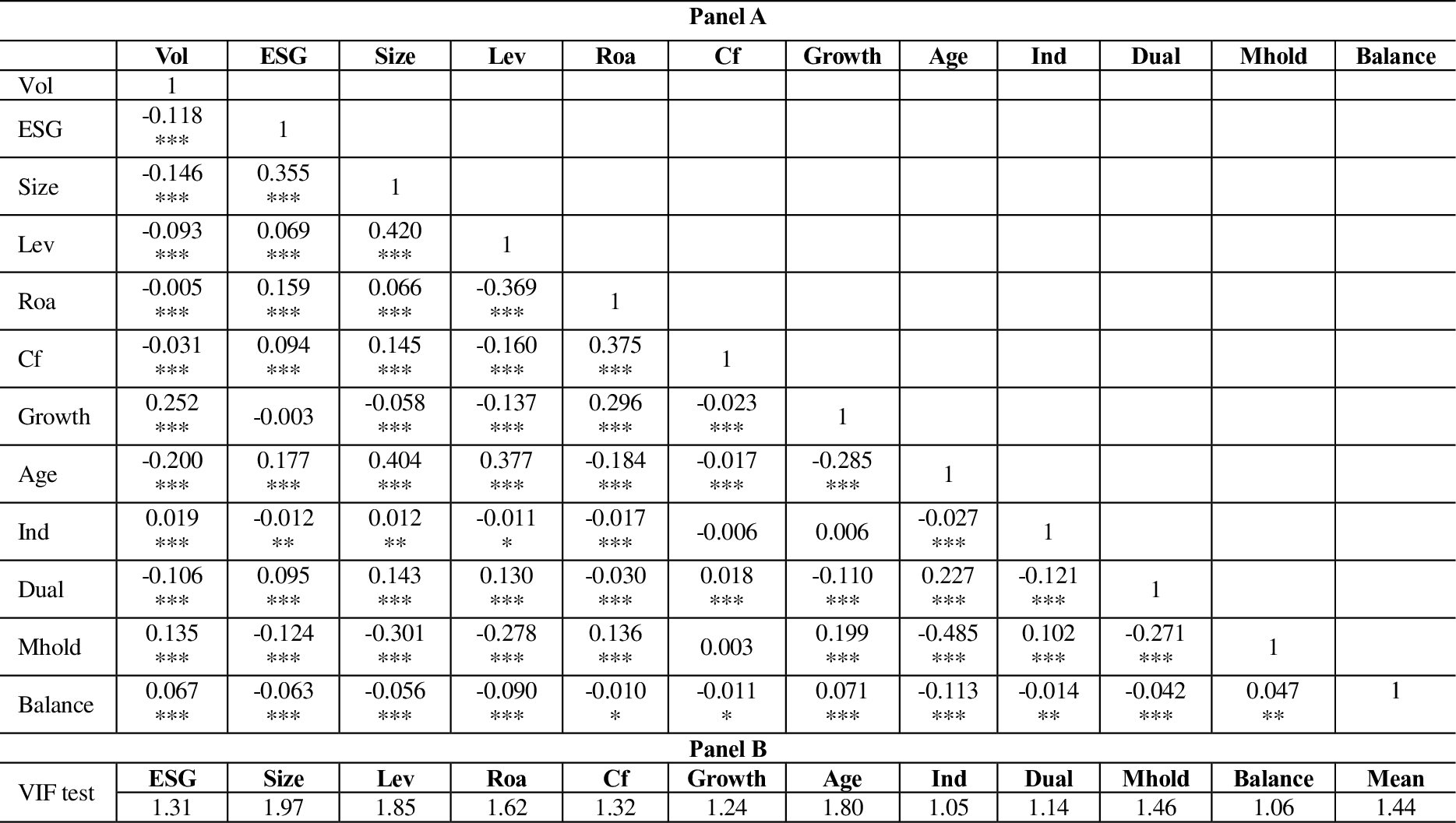
**

**Table 4. Baseline regression test results.**


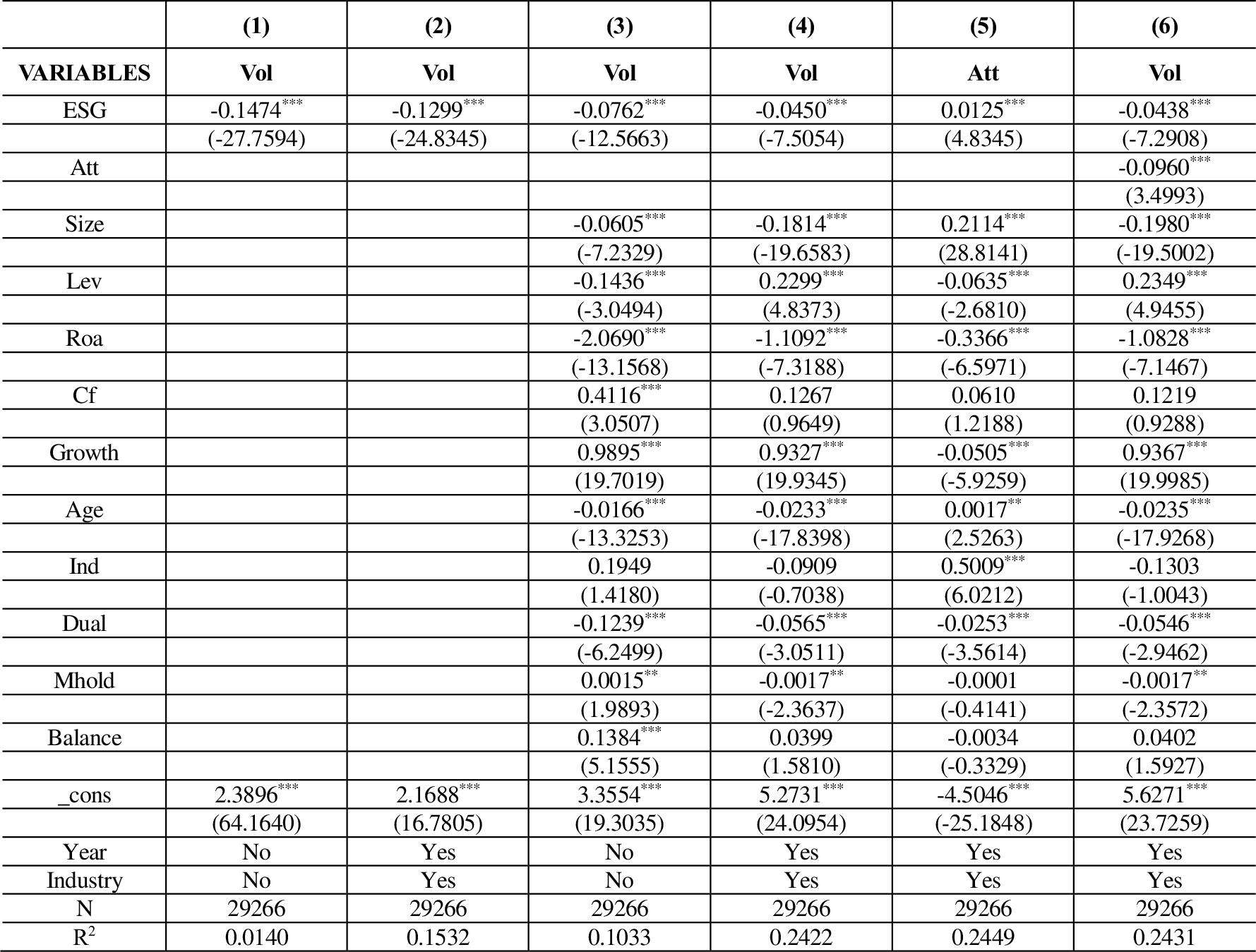


**Table 5. Robustness test results.**


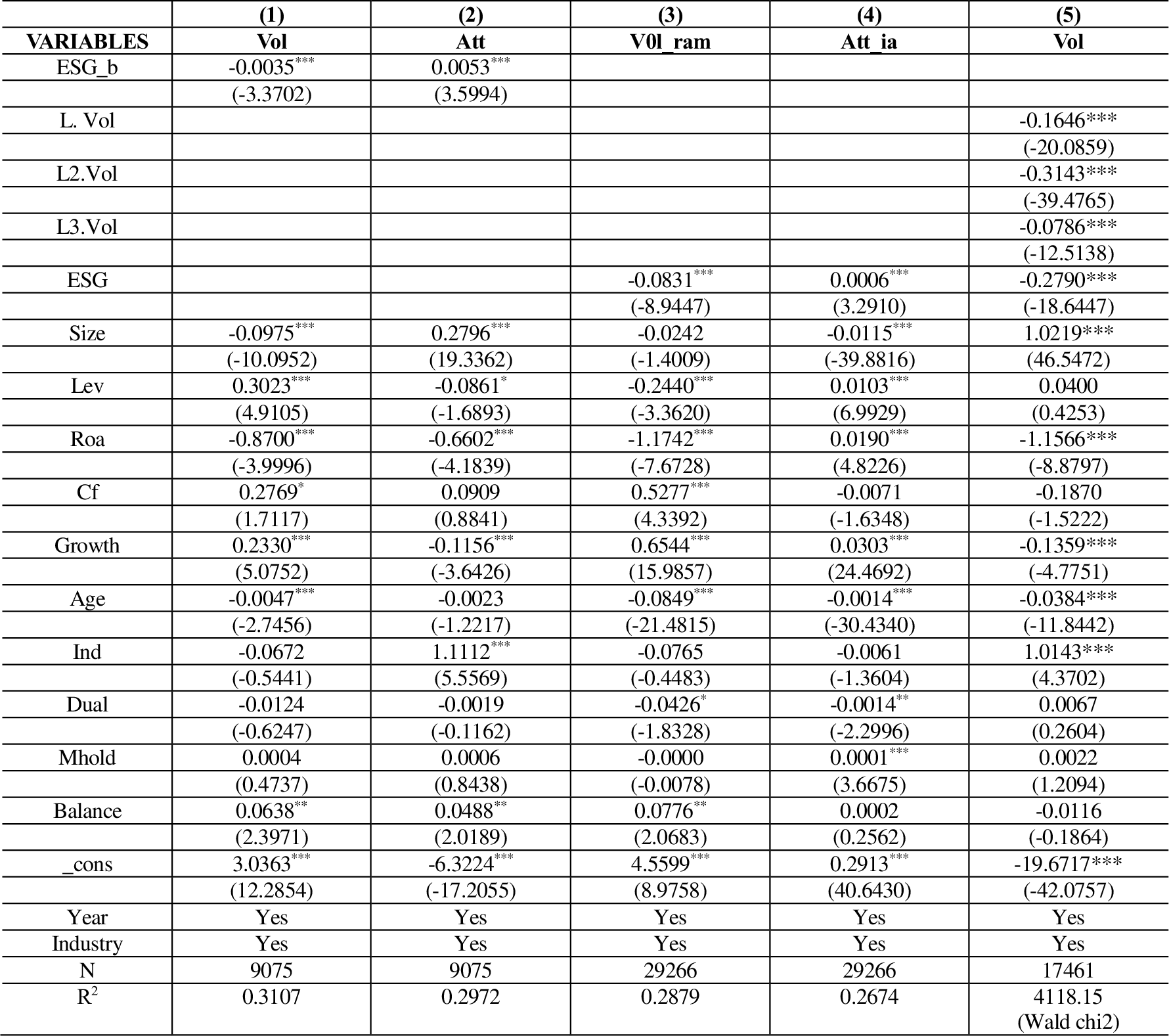


**Table 6. Endogeneity test results.**


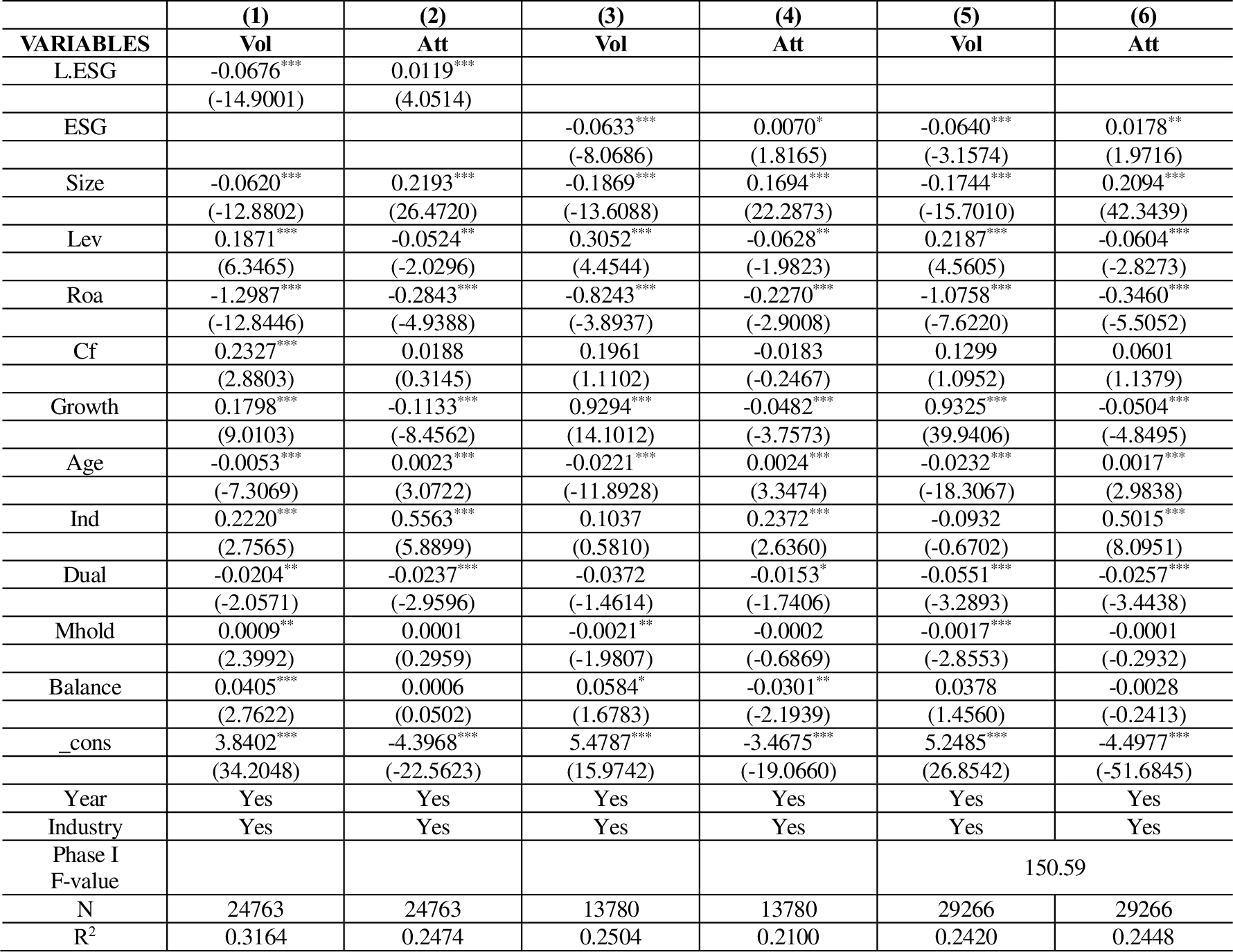


**Table 7. Heterogeneity analysis results.**


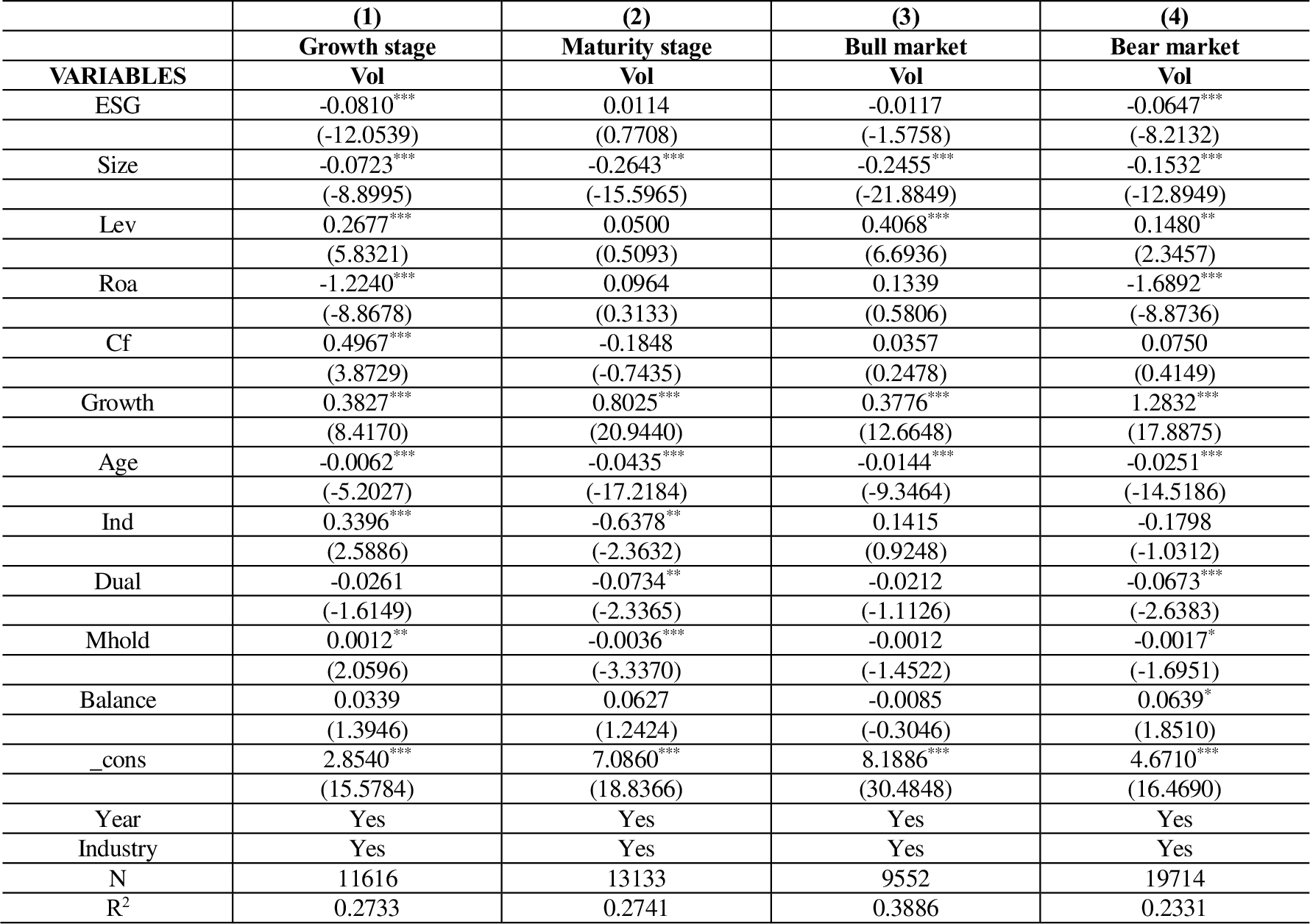

Supplement: S1 Table — (DOCX) [file pone.0307535.s001.docx]
