# Supplementary material for: Can good ESG performance of listed companies reduce abnormal stock price volatility? Mediation effects based on investor attention
Source: PLoS One. 2024 Sep 6;19(9):e0307535. doi: 10.1371/journal.pone.0307535 (PMC11379154; doi:10.1371/journal.pone.0307535)
Supplement: Attachment — Submitted filename: PONE-D-23-38843.docx [file pone.0307535.s002.docx]

Review Report for Manuscript ID: PONE-D-23-38843. “Can good ESG performance of listed companies reduce abnormal stock price fluctuations?--Empirical evidence from China” I applaud the authors for their efforts in crafting this article. However, there are a number of issues with this paper that need to be revised. These concerns are delineated as follows:

1 In line 345, there is a problem with the three-line table setting in Table 2.

2 In line 302, there are problems with literature citations, and the same problem also exists in many places.

3 In lines 323 – 332, the authors do not explain in detail the meaning of the parts of the formula.

4 Suppose a problem with H2 setup. This article focuses on the relationship between ESG performance and abnormal stock price fluctuations. Why do we still need to explore the relationship between ESG performance and investor attention? The authors set investor attention as an intermediary variable, but should also combine H2 and H3.

5 In the heterogeneity analysis section, why is the total sample volume of the enterprise life cycle and the bull and bear market inconsistent?

6 In the literature review section, the author did not do a good job of literature together, many places directly put the literature, so we need to carry out some sorting.

**My Recommendation**: Major Revision
