# Supplementary material for: Can good ESG performance of listed companies reduce abnormal stock price volatility? Mediation effects based on investor attention
Source: PLoS One. 2024 Sep 6;19(9):e0307535. doi: 10.1371/journal.pone.0307535 (PMC11379154; doi:10.1371/journal.pone.0307535)
Supplement: Attachment — Submitted filename: PONE-D-23-38843.docx [file pone.0307535.s003.docx]

Can good ESG performance of listed companies reduce abnormal stock price fluctuations? Empirical evidence from China

PONE-D-23-38843

Here are some suggested comments for the authors:

1- Authors need to theoretically explain the nature of the association between ESG and the abnormal stock price volatility. (Introduction section)
2- Authors need to theoretically explain the nature of the association between ESG and the investor. (Introduction section)
3- Introduction is too short. Additional empirical studies are recommended to strengthen the introduction section.
4- Research contributions need to be explained more clearly, and precisely (what about the mediation).
5- I think you need to add a sentence like this: (The remainder of this paper is structured as follows. Section 1 Literature Review; Section 2…….etc. Please add this sentence before Literature Review section.
6- Please provide a correlation matrix table, like the Pearson correlation matrix and the Variance Inflation Factor (VIF). You can add the correlation matrix table after the descriptive statistics section.
7- How investor attention (Att) is measured. The way that the author explains the variable is not clear. Please explain the investor attention measurement. (Mediating variable: investor attention (Att) section).
8- Finally, I recommend adding more method in the robustness check section; the author(s) are highly recommended to employ an optimal technique like GMM.

All the best.
