# Supplementary material for: Can good ESG performance of listed companies reduce abnormal stock price volatility? Mediation effects based on investor attention
Source: PLoS One. 2024 Sep 6;19(9):e0307535. doi: 10.1371/journal.pone.0307535 (PMC11379154; doi:10.1371/journal.pone.0307535)
Supplement: Attachment — Submitted filename: Response to Reviewers.docx [file pone.0307535.s004.docx]

**Response to Reviewers' Comments on Manuscript [PONE-D-23-38843]**

Dear Dr. María del Carmen Valls Martínez,

I am writing to submit our revised manuscript titled "Can good ESG performance of listed companies reduce abnormal stock price fluctuations?----Mediation effects based on investor concerns" for PLOS ONE, bearing the manuscript ID [PONE-D-23-38843]. We are deeply grateful to you and the reviewers for your insightful comments and suggestions, which have significantly improved the quality of our paper. In the revised manuscript, we have closely adhered to the PLOS ONE formatting style, and all data tables have been organized into a separate document as supporting information. We have meticulously addressed each comment and suggestion made by the reviewers. Below is a summary of how we responded to each point.

**Reviewer #1:**

1.Originality: Does the paper contain new and significant information adequate to justify publication?

Response:We have substantially revised the introduction section to enhance its clarity and relevance. The paper's significant contribution lies in bridging a gap in the existing literature, offering insights into solving global environmental and social crises, and aiding in the stabilization of the capital markets. This paper contains sufficient novel information to warrant its publication. We will detail the specific amendments made in our responses to the subsequent points.

Comment 1: It is a good initiative by the authors which to examine the ESG performance of listed companies with stock price which is one of the current issues. However, the intention of this study is not clearly outline. Author should explain and discuss the so what question. What is the urgency of this study?

Response: In response to Comment 1, we have thoroughly revised the introduction of our manuscript to more clearly articulate the significance and urgency of this study. Firstly, we introduce the research background, highlighting the global challenges of environmental and social responsibility. The growing focus on ESG investment worldwide, along with investors' heightened concern over corporate ESG performance, underscores the relevance of our study. Secondly, we address the gap in existing literature. There are limited studies exploring the impact of corporate ESG performance on abnormal stock price volatility, and existing research shows significant divergence in conclusions. Few studies delve into how investor concern influences the relationship between ESG performance and abnormal stock price volatility. Our study seeks to explore this relationship from the perspective of investor concern, thereby filling a critical gap in the literature. This offers valuable theoretical insights and practical implications for investors, corporate decision-makers, and policymakers. Given the current fragile state of global economic recovery and the intensified volatility of stock prices, particularly in the Chinese market known for its dramatic fluctuations, this research is both urgent and necessary. It aims to contribute to the sustainable development of companies and the stability of the capital market. The revised introduction now clearly outlines the study's intentions, its urgency, and its theoretical and practical significance, thus highlighting the necessity and importance of this research.

Comment 2: please make a stronger case as to why your paper is needed and how you contribute to current academic discussion. Although authors have presented the gap in the introduction, but it is not sufficient. You should be more convincing and using the current literature review to indicate the gap that needs to be closed. You have to explain why the gap matters. Why is the gap a real concern? What will go wrong if the gap is unaddressed? The authors need to explain better what is the main puzzle their research is addressing

Response: In response to Comment 2, our revised manuscript elaborates on the necessity and contribution of this study, drawing on extensive literature review and theoretical analysis. We acknowledge the existing inconsistencies in research findings regarding the impact of corporate ESG performance on abnormal stock price volatility. Some studies suggest that strong ESG performance significantly reduces abnormal stock price volatility, while others indicate the opposite. Furthermore, while better ESG performance of listed firms tends to attract more investor attention, this increased attention can paradoxically exacerbate stock price volatility, potentially leading to a "crash effect of attention." This dichotomy underscores the critical gap our study aims to bridge.

Our revised introduction emphasizes the main research question: Does exemplary ESG performance by listed companies induce abnormal share price volatility through heightened investor attention? What is the role of investor attention in this context? And does this relationship vary depending on the firm's life cycle or market state? By addressing these questions, our research seeks to clarify the ambiguous effects of ESG performance on stock volatility. We aim to contribute significantly to the academic discourse by dissecting the nuanced interplay between ESG performance, investor attention, and stock price volatility. This exploration is crucial, as neglecting this gap might lead to an inadequate understanding of the factors influencing market stability and corporate responsibility, thereby impeding effective policy-making and investment strategies.

Comment 3: Authors has highlighted one past empirical study. However, authors should provide more elaboration and highlight deficiencies or gaps in the existing literature. The authors should offer a more comprehensive analysis of the limitations present in previous studies to establish the novelty and unique contributions of their study, especially the theoretical aspect.

Response: In response to Comment 3, we have substantially expanded our discussion of previous empirical studies in the revised manuscript. We conducted a detailed comparison of the existing literature exploring the relationship between ESG and stock price volatility. This comparison reveals a significant divergence in findings, highlighting a crucial gap: the lack of research examining the impact of ESG on stock price volatility from the perspective of investor attention.

Our comprehensive analysis in the revised manuscript identifies and elaborates on these limitations. We underscore the absence of an integrative approach that combines ESG performance assessment with the dynamics of investor attention and its subsequent effect on stock price volatility. Our study uniquely contributes to this area by not only investigating the direct relationship between ESG performance and abnormal stock price volatility but also exploring the mediating role of investor attention. This approach bridges the identified gaps and adds a novel dimension to the existing body of literature.

We believe that our study's focus on the interplay between ESG performance and investor attention represents a significant advancement in the theoretical understanding of stock market dynamics. It addresses the previously overlooked aspects of how investor perceptions and reactions to ESG performance can influence market behavior, thus offering a more holistic view of the factors reducing stock price volatility.

Comment 4: I would like to suggest author to revise the introduction which can consider following flow of the introduction and addressing the following questions: (1) Briefly describe and illustrate the current issue. (2) Why is such a study with proposed research gaps important? (3) How does this research gap relate to the current issue? (4) Why is such an underexplored piece of work important to be tested in your study? (5) Are there any similar studies conducted in the past? (6) What is the uniqueness of this study compared to past empirical studies? (7) What are the research objectives? (8) What are the contributions of the study?

Response: In response to the reviewers' suggestions, we have revised the introduction to reflect the suggested structure. We briefly describe the current global crisis and highlight the importance of ESG concepts, illustrating how firms' ESG performance impacts stock price volatility. Our comparison of literature on the ESG-stock price volatility relationship reveals significant divergence in findings. The importance of investigating this discrepancy lies in its relevance to the increasingly volatile global stock markets. This research gap is closely related to the current issue, and addressing it will lead to a deeper understanding of the intrinsic mechanisms by which firms' ESG performance affects stock price volatility. Such an under-explored area of research is crucial in addressing the current global crisis and abnormal stock price volatility, forming the core of this study. While there are a few past studies examining the relationship between ESG and stock price volatility, their conclusions are inconsistent, and few have examined how investor attention influences the relationship between firms' ESG performance and abnormal stock price volatility. Compared to previous empirical studies, the uniqueness of this study lies in using investor attention as a mediating variable to reveal whether good ESG performance of listed companies can mitigate abnormal stock price volatility. It is committed to providing empirical insights for investors in stock selection and corporate decision-makers in ESG investment. Utilizing a unique dataset of Chinese listed companies, this study empirically explores the relationship between corporate ESG performance and abnormal stock price volatility, examining the mediating role of investor attention, aiming to fill the gap in previous literature research. Through this study, it aims to provide theoretical guidance and practical experience for investors, corporate decision-makers, and policymakers. This study points out three potential contributions, which fill gaps in the existing literature; enrich research on the economic consequences of corporate ESG performance and factors affecting stock price volatility; and offer empirical data for listed companies to assess the value of corporate ESG and for securities regulators to foster the healthy development of the capital market.

2.Relationship to Literature: Does the paper demonstrate an adequate understanding of the relevant literature in the field and cite an appropriate range of literature sources? Is any significant work ignored?

Response:We have extensively revised the literature review to ensure a comprehensive examination of the relevant literature in the field of ESG and stock price volatility. We meticulously cited relevant sources to ensure that no significant work is omitted. The specific revisions are detailed in the responses to the points below.

Comment 5: Literature review is required. Authors should bear in mind that a good literature review is a comprehensive and well-structured synthesis of existing scholarly works and research findings on a specific topic. It involves identifying, analyzing, and evaluating relevant sources to provide a critical overview of the current knowledge and gaps in the field. Author should provide a critical and comprehensive review, before embarking to any study method.

Response:We have substantially enhanced the literature review by including a wide range of relevant literature and systematically evaluating these sources. Initially, we comprehensively reviewed the literature on the economic consequences of ESG performance, addressing the impact of ESG performance on corporate financing, investment, innovation, financial performance, and operational efficiency. Subsequently, we synthesized the literature on the relationship between corporate ESG performance and abnormal stock price volatility, examining perspectives from governance (G), environmental (E), social (S), and comprehensive ESG aspects. We found that most existing studies focus on the governance aspect's impact on stock price volatility, with limited literature investigating the comprehensive ESG perspective's impact and noted significant divergence in research conclusions. Furthermore, analyzing the literature on the impact of investor concern on stock price volatility, we identified two different schools of thought in academia: the "deterioration theory" and the "stabilization theory." However, almost no literature examines the mechanism of investor concern in the relationship between ESG performance and abnormal stock price volatility. Finally, the existing literature mainly focuses on the impact of ESG performance on daily corporate operations with little attention to its effect on abnormal stock price volatility, resulting in divergent findings. Moreover, from the perspective of the mediating effect of investor concern, there is almost no literature on the impact of ESG performance on abnormal stock price volatility. This gap demonstrates divergences and opportunities in the existing literature. Our paper empirically investigates the impact of corporate ESG performance on abnormal stock price volatility using empirical data from Chinese A-share listed companies in Shanghai and Shenzhen, focusing on analyzing the mechanism of investor concern in this relationship, which holds theoretical and practical research value.

Comment 6:Hypotheses can be more straightforward, especially the mediating path.

Response:We have refined the paper's hypotheses, particularly the mediated pathway hypothesis, for clearer and more direct presentation.

3.Methodology: Is the paper's argument built on an appropriate base of theory, concepts, or other ideas? Has the research or equivalent intellectual work on which the paper is based been well designed? Are the methods employed appropriate?

Response: In response to the reviewers' comments on methodology, we refined the theoretical framework to ensure a sound research design. The research methodology utilizes an empirical research approach that is appropriate for the objectives of the dissertation study. The specific modifications are shown in the following points.

Comment 7:I am suggesting authors to revise the title for definitions of variables to operationalization of the variables.

Response:We have revised the variable titles to more accurately reflect their nature and the subsequent sections detail their operationalization.

Comment 8: Including control variables in the study is indeed a good practice as they can help account for potential confounding factors that may influence the results. To improve the paper, it is important to include control variables and provide reasonable justifications for their inclusion.

Response:We have revised the control variables section, providing a rationale for their selection based on existing literature, and because all these control variables affect stock price volatility.

Comment 9: The authors should discuss the generalizability and representativeness of their sample in relation to the target population. Authors need to clearly explain how the chosen sample is intended to be representative of and reflective of the larger population. Any strategies employed to ensure a diverse and inclusive sample should also be highlighted. This will increase the credibility of the research findings and help readers understand the extent to which the results can be generalized to the broader population.

Response:The sample companies in this study are all listed companies in China's Shanghai and Shenzhen A-shares from 2011 to 2020. Since the starting year of ESG rating data is 2010 and updated to 2021, we select the data of listed companies for the 10 years from 2011 to 2020. The data are screened in accordance with the convention of existing literature research, and the data for continuous variables are also reduced-tailed at 1% and 99% to prevent the effect of extreme values of data. The data of the selected sample companies are comprehensive and representative, and the study's results are credible and can be generalized to a wider population.

Comment 10：Is this data sufficient to draw a robust finding? Why was this secondary data used? Any other types of data could be used?

Response: The data in this paper are all from authoritative databases, with ESG ratings and investor focus data from the Wind database, and the rest from the Cathay Pacific CSMAR database. This dataset is robust and sufficient for drawing reliable conclusions. The ESG data are adopted from the Huazheng index, covering all A-share listed companies with the most comprehensive data, widely recognized in industry and academia. In the robustness test, other types of data are used to verify the reliability of the conclusions.

Comment 11：The authors should provide adequate justification for the use of the software in this study. They should explain why this particular software was chosen and how it aligns with the research objectives. By elaborating on the software's specific functionalities and how it supports the analysis of the data, the authors can enhance the validity and reliability of their findings.

Response:We have added a note in the text that this paper uses Stata17 software for statistical processing of data, which is commonly used in empirical economics research. It is powerful and can guarantee the validity and reliability of the research results.

5.Practicality and/or Research implications: Does the paper identify clearly any implications for practice and/or further research? Are these implications consistent with the findings and conclusions of the

Response:We carefully reviewed and revised the manuscript to ensure that implications for practice and future research were clearly identified. These implications are consistent with the findings of the study. See the responses to the points below for specific revisions.

Comment 12: The ending part of the manuscript is not well written.

Response:We acknowledge the reviewers' comments and have revised the concluding section of the paper to enhance the logical coherence of the article.

Comment 13: I would suggest author to structure their conclusion part of the manuscript as follows: (1) Discussion, (2) theoretical Implications, (3) practical implications/policy implications, (4) Limitations and Future Research Recommendations, and (5) Conclusion.

Response:Thank you for your suggestions. We have reorganized the conclusion section of the paper according to the recommended format, including discussion, theoretical implications, practical implications/policy recommendations, research limitations and recommendations for future research, and conclusions.

Comment 14: Should have a standalone section for discussion, the authors need to ensure that the key findings or scale development are discussed. The discussion section is where you delve into the meaning, importance, and relevance of your results. It should focus on explaining and evaluating what you found, showing how it relates to your literature review and research questions, and making an argument in support of your overall conclusion. Limited citations. Please cite more to support the discussion.

Response:We have added a separate discussion section that focuses on explaining and evaluating the paper's research findings, showing how it relates to the literature review and research questions. And more literature citations were added to support the discussion.

Comment 15: For theoretical implications. How can you imply from the findings? This section should discuss the implications of the study's findings and how they contribute to the existing theoretical knowledge. Summarize the key findings and their relevance to the existing theoretical frameworks or models. Analyze how the findings align with or challenge current theoretical perspectives and concepts related to all the key concepts of this study. Discuss any theoretical insights or advancements that the study provides and highlight how the findings contribute to a deeper understanding of the research area.

Response:We have revised the theoretical implications section, and the findings of this paper make up for the shortcomings of previous studies in the literature by further explaining the mechanism of the impact of ESG performance on abnormal stock price volatility, which contributes to the existing theoretical field.

Comment 16: Should have a standalone section for practical implications: the authors should provide valuable insights based on current practices and policies, supported by evidence from their research. To strengthen the practical implications, it is crucial to reference specific findings, data, or examples that demonstrate the validity and reliability of the recommendations. By incorporating this approach, the authors can offer concrete and actionable suggestions that have a solid grounding in their research findings.

Response:We have included practical advice as a separate section, which provides some valuable practical operational advice to listed companies, investors and governments, which is supported by evidence from our research.

Comment 17: Standalone section for limitation and future research recommendation, it is not sufficiently written. Please reconsider and identify limitations of the study.

Response:We have added independent study limitations and recommendations for future research, and we provide a comprehensive discussion of the study's limitations and specific recommendations for future research.

6. Quality of Communication: Does the paper clearly express its case, measured against the technical language of the field and the expected knowledge of the journal's readership? Has attention been paid to the clarity of expression and readability, such as sentence structure, jargon use, acronyms, etc.

Proofreading is required. Cite more recent studies. Relook into the structure of the manuscript.

Response:We appreciate the reviewer’s feedback on the quality of communication. We have carefully proofread the manuscript to ensure clarity of expression and readability, addressing issues such as sentence structure, jargon use, and acronyms. We have also incorporated more recent studies in the relevant sections to strengthen the literature review and improve the currency of the paper. Additionally, we have re-evaluated the structure of the manuscript to enhance its coherence and organization. Thank you for these valuable suggestions, and we believe the manuscript has been significantly improved in these aspects.

**Reviewer #2:**

Comment 1：In line 345, there is a problem with the three-line table setting in Table 2.

Response:We have fixed the three-row table setup issue in Table 2 on the original line 345 to ensure accurate table formatting. And we've fixed the setup issues that also existed with the other tables in the article.

Comment 2：In line 302, there are problems with literature citations, and the same problem also exists in many places.

Response:We have corrected the bibliographic citation problem in the original line 302 and have scrutinized the entire text for the same bibliographic citation problem that exists elsewhere, all of which have been corrected.

Comment 3：In lines 323–332, the authors do not explain in detail the meaning of the parts of the formula.

Response:Below the formula, we have added a detailed explanation of the meaning of each part of the formula to ensure that the reader understands the formula.

Comment 4：Suppose a problem with H2 setup. This article focuses on the relationship between ESG performance and abnormal stock price fluctuations. Why do we still need to explore the relationship between ESG performance and investor attention? The authors set investor attention as an intermediary variable, but should also combine H2 and H3.

Response:This paper focuses on the relationship between ESG performance and abnormal stock price volatility, and takes investor attention as a mediating variable to investigate the mediating role of investor attention between corporate ESG performance and abnormal stock price volatility. Therefore, we integrate the original hypotheses of H2 and H3 and propose a new hypothesis H2: Good ESG performance of listed companies can significantly enhance investor attention, and investor attention plays a mediating role in reducing abnormal stock price volatility. Moreover, in order to reflect the use of investor attention as a mediating variable, the subtitle of the article is changed to "Mediation effect based on investor attention".

Comment 5：In the heterogeneity analysis section, why is the total sample volume of the enterprise life cycle and the bull and bear market inconsistent?

Response: Dickinson (2011) divides the life cycle of enterprises into five stages: start-up, growth, maturity, turbulence and recession, and determines the life cycle stage of enterprises through different combinations of three indicators: operating cash flow, investment cash flow and financing cash flow. Considering China's requirements for listed companies, companies that can pass the A-share listing audit basically go through the start-up period, and companies in the decline period are basically in the delisted state, so this paper combines the start-up period and the growth period into the growth period, and samples with close characteristics of companies in the maturity period and the turbulence period into the maturity period, and doesn't take into account the companies in the decline period, and therefore, the total sample size of the life cycle of the enterprise is smaller compared to that of the bull market and the bear market.

Comment 6：In the literature review section, the author did not do a good job of literature together, many places directly put the literature, so we need to carry out some sorting.

Response: We have revamped the literature review section and organized the literature according to a certain logic. First, the literature on the economic consequences of ESG performance is comprehensively organized, discussing the impact of ESG performance on corporate financing, investment, innovation, financial performance, and operational efficiency. Second, the literature on the relationship between firms' ESG performance and abnormal stock price volatility is synthesized, respectively, from G, E, S, and comprehensive ESG perspectives. Third, we analyze the literature on the impact of investor concerns on stock price volatility, and find that there are two different schools of thought in the academic community: the "deterioration theory" and the "stabilization theory", but there is almost no literature that examines the mechanism of investor concerns on the relationship between ESG performance and abnormal stock price volatility. The literature on ESG performance and stock price volatility has not been studied in any way. Finally, in the literature review, the existing literature mainly focuses on the impact of ESG performance on the daily operation of enterprises, and pays little attention to the impact of ESG performance on abnormal stock price volatility, and the findings of the research are quite divergent. And from the perspective of the mediating effect that investors are concerned about, there is almost no literature on the impact of ESG performance on abnormal stock price volatility. By organizing the literature in an organized manner, we find the differences and gaps in the existing literature research, which highlights the value of the research in this paper.

**Reviewer #3:**

Comment ：Incorporate the suggested comments in the file attached. You can slight look for new literature in your perspective. There is very good research on the emerging economies and look for the covid-19 based papers they will be good ground as well.

Response: We have carefully reviewed the attached file and incorporated the suggested comments into the manuscript. Additionally, we have conducted a search for new literature, particularly focusing on research related to emerging economies and the impact of COVID-19. The new literature has been integrated into our perspective to enhance the relevance and depth of our analysis.

**Reviewer #4:**

Comment 1：Authors need to theoretically explain the nature of the association between ESG and the abnormal stock price volatility. (Introduction section)

Response: Thanks to your suggestion, we have revised the introduction section by adding a theoretical explanation of the correlation between ESG and abnormal stock price volatility to strengthen the theoretical foundation of the text.

Comment 2：Authors need to theoretically explain the nature of the association between ESG and the investor. (Introduction section)

Response: We revise the introduction section by adding theoretical explanations of the correlation between ESG and investors, including signaling theory, reputation theory, and limited rationality theory.

Comment 3：Introduction is too short. Additional empirical studies are recommended to strengthen the introduction section.

Response: We have expanded the introductory section by adding an in-depth analysis of the research context and citing more empirical studies from the literature to flesh out the introductory section.

Comment 4：Research contributions need to be explained more clearly, and precisely (what about the mediation).

Response: We have revised the Research Contributions section by expanding the research contributions from the original two points to three, which provides a clearer and more accurate explanation of the research contributions, particularly the role of the mediating variables.

Comment 5：I think you need to add a sentence like this: (The remainder of this paper is structured as follows. Section 1 Literature Review; Section 2…….etc. Please add this sentence before Literature Review section.

Response: Thanks to the suggestion, we have added similar sentences before the literature review section to better guide the reader through the article structure.

Comment 6：Please provide a correlation matrix table, like the Pearson correlation matrix and the Variance Inflation Factor (VIF). You can add the correlation matrix table after the descriptive statistics section.

Response: We have added a table of correlation matrices after the descriptive statistics section, which includes the Pearson correlation matrix and the VIF.

Comment 7：How investor attention (Att) is measured. The way that the author explains the variable is not clear. Please explain the investor attention measurement. (Mediating variable: investor attention (Att) section).

Response: We have provided a clearer explanation of how investor focus is measured to ensure the reader's understanding of the variable.

Comment 8: Finally, I recommend adding more method in the robustness check section; the author(s) are highly recommended to employ an optimal technique like GMM.

Response: Thanks to the suggestion, we have added the GMM optimization technique to the robustness test section and the conclusions still hold, proving the robustness of the results of this study.

**Reviewer #5:**

Thanks for the submission.While I applaud the authors' efforts, the manuscript does not contribute novelty in the research findings.The manuscript's contribution is already existed in latest published research.

Response: Thank you for your review. Although there is a small amount of literature examining the relationship between ESG and abnormal stock price volatility, there is no unified research conclusion. Few literatures have examined the mediating effect of investor concern between firms' ESG performance and abnormal stock price volatility from the perspective of studying investor concern. Compared with previous empirical studies, the uniqueness of this study is to use investor concern as a mediating variable to reveal whether good ESG performance of listed companies can mitigate abnormal stock price volatility. The findings and heterogeneity analysis of this study can provide theoretical guidance and practical experience for investors, corporate decision makers and policy makers, which is the main contribution of this study.

We believe that these revisions significantly enhance the manuscript in clarity, significance, and originality. Thank you for the opportunity to revise our paper based on your valuable feedback. Additional minor modifications have been made to further improve readability, all of which are highlighted in blue for easy identification. We hope our revisions meet the reviewers' expectations and look forward to the potential publication in PLOS ONE. We appreciate the chance to refine our manuscript with constructive feedback.

Thank you for your time and consideration.

Sincerely,

Fengju Wu

E-mail: wufengju@naujsc.edu.cn
