# Supplementary material for: Can good ESG performance of listed companies reduce abnormal stock price volatility? Mediation effects based on investor attention
Source: PLoS One. 2024 Sep 6;19(9):e0307535. doi: 10.1371/journal.pone.0307535 (PMC11379154; doi:10.1371/journal.pone.0307535)
Supplement: Attachment — Submitted filename: Reviewe.docx [file pone.0307535.s006.docx]

The paper has been greatly improved, but there are still a few minor changes that need to be made.

1．The theoretical analysis in the third section lacks sufficient literature support.

2．The title of Sections 7.1 is not clear enough and does not match the main title of Chapter 7.

3．The discussion in sections 7.1 did not delve deeply into the existing literature. The author mentioned literature with different results, which can be analyzed and compared in more detail. Also, there is an error in the citation of the literature on line 605, and I cannot see which one it is.
