# Supplementary material for: Can good ESG performance of listed companies reduce abnormal stock price volatility? Mediation effects based on investor attention
Source: PLoS One. 2024 Sep 6;19(9):e0307535. doi: 10.1371/journal.pone.0307535 (PMC11379154; doi:10.1371/journal.pone.0307535)
Supplement: Attachment — Submitted filename: Response to Reviewers-2.docx [file pone.0307535.s007.docx]

**Response to Reviewers' Comments on Manuscript [PONE-D-23-38843]**

Dear Dr. Valls Martínez,

I am writing to submit our revised manuscript titled "Can good ESG performance of listed companies reduce abnormal stock price fluctuations?Mediation effects based on investor concerns" for PLOS ONE, bearing the manuscript ID [PONE-D-23-38843]. We are deeply grateful to you and the reviewers for your insightful comments and suggestions, which have significantly improved the quality of our paper. We have meticulously addressed each comment and suggestion made by the reviewers. Below is a summary of how we responded to each point.

Comment 1: The theoretical analysis in the third section lacks sufficient literature support.

Response: Thank you for your insightful comments. To address this issue, I have conducted a comprehensive review of the relevant literature and added several references to support the theoretical analysis. Specifically, 3-4 articles were added to Section 3.1, and 6-7 articles were added to Section 3.2. These additional references have enhanced the depth and breadth of the theoretical analysis, ensuring that the development of theoretical analysis and research hypotheses is well-supported by the literature.

Comment 2: The title of Sections 7.1 is not clear enough and does not match the main title of Chapter 7.

Response: Thank you for your thorough review. I have revised the title of Section 7.1 to "Conclusions and Discussion" to align it with the main title of Chapter 7, which is "Conclusions and Recommendations." Section 7.1 serves as the first part of this chapter.

Comment 3: The discussion in sections 7.1 did not delve deeply into the existing literature. The author mentioned literature with different results, which can be analyzed and compared in more detail. Also, there is an error in the citation of the literature on line 605, and I cannot see which one it is.

Response: Thank you for your pertinent comments. In order to make a more in-depth comparative analysis of the existing literature, I firstly reviewed the literature with different viewpoints in the 2 Literature Review section and added 2 additional documents, and then analyzed and compared the literature with different viewpoints in detail in section 7.1. After discussion and analysis, it is found that the findings of this paper validate the mainstream literature. And the mediation effect test and heterogeneity analysis make up the gap in the existing literature, highlighting the value of the research in this paper.

In addition, you mentioned that there is an error in the literature on line 605 can not find this literature, probably because the original volume number 19 is not written clearly. This literature [3] is real at https://doi.org/10.1108/JFRA-01-2020-0020. The volume number of reference [3] has now been added.

**Reviewer #4:**

We have made serious revisions to each of the comments of reviewer #4 last time. Now we have slightly revised them on the basis of the original ones, mainly the first point and the seventh point. The specific responses are as follows.

Comment 1：Authors need to theoretically explain the nature of the association between ESG and the abnormal stock price volatility. (Introduction section)

Response: Thanks to your suggestion, we have revised the introduction section by adding risk management theories and deleting the original theories to better explain the correlation between ESG and abnormal stock price volatility.

Comment 2：Authors need to theoretically explain the nature of the association between ESG and the investor. (Introduction section)

Response: We have revised the introduction section by adding signaling theory, reputation theory, and limited rationality theory to better explain the correlation between ESG and investors.

Comment 3：Introduction is too short. Additional empirical studies are recommended to strengthen the introduction section.

Response: We have expanded the introductory section by adding an in-depth analysis of the research context and citing more empirical studies from the literature to flesh out the introductory section.

Comment 4：Research contributions need to be explained more clearly, and precisely (what about the mediation).

Response: We have revised the Research Contributions section by expanding the research contributions from the original two points to three, which provides a clearer and more accurate explanation of the research contributions, particularly the role of the mediating variables.

Comment 5：I think you need to add a sentence like this: (The remainder of this paper is structured as follows. Section 1 Literature Review; Section 2…….etc. Please add this sentence before Literature Review section.

Response: Thanks to the suggestion, we have added similar sentences before the literature review section to better guide the reader through the article structure.

Comment 6：Please provide a correlation matrix table, like the Pearson correlation matrix and the Variance Inflation Factor (VIF). You can add the correlation matrix table after the descriptive statistics section.

Response: We have added a table of correlation matrices after the descriptive statistics section, which includes the Pearson correlation matrix and the VIF.

Comment 7：How investor attention (Att) is measured. The way that the author explains the variable is not clear. Please explain the investor attention measurement. (Mediating variable: investor attention (Att) section).

Response: We have provided a clearer explanation of how investor focus is measured to ensure the reader's understanding of the variable.

Comment 8: Finally, I recommend adding more method in the robustness check section; the author(s) are highly recommended to employ an optimal technique like GMM.

Response: Thanks to the suggestion, we have added the GMM optimization technique to the robustness test section and the conclusions still hold, proving the robustness of the results of this study.

We believe that these revisions have greatly improved the clarity, importance and originality of the manuscript. Thank you for giving us this opportunity to revise our paper based on your valuable comments. In order to further improve readability, we have made some minor modifications, such as slightly modifying suggestions and future research prospects based on the discussion content, adding references and adjusting the format and numbering, and proofreading the entire article. All these modifications are highlighted in red for easy identification. We hope that our revisions meet the expectations of the reviewers and look forward to the publication of the paper in PLOS ONE. We appreciate the opportunity to improve our manuscript through constructive feedback.

Thank you for your time and consideration.

Sincerely,

Fengju Wu

E-mail: wufengju@naujsc.edu.cn
