# Supplementary material for: Can good ESG performance of listed companies reduce abnormal stock price volatility? Mediation effects based on investor attention
Source: PLoS One. 2024 Sep 6;19(9):e0307535. doi: 10.1371/journal.pone.0307535 (PMC11379154; doi:10.1371/journal.pone.0307535)
Supplement: Attachment — Submitted filename: Response to Reviewers.docx [file pone.0307535.s008.docx]

Dear reviewers,

We are very grateful for your constructive comments and suggestions for our manuscript. Your comments are very valuable and helpful for improving our manuscript. In the following, the responses to all the comments are provided one by one. Furthermore, we have carefully revised and polished the entire manuscript to enhance its readability. All changes are marked in red.

We have tried our best to make all the revisions clear, and we hope that the revised manuscript can satisfy the requirements for publication. Below are our main responses to the reviewers' comments.

**Response to the comments of**

**Reviewer #6**

Comment 1: Line 244: “information” should be “Information”

Line 311: put a space between al. and reference, and also between reference and “to” (Zhang et al.[30]to)

Line 389: please give a space between Jiang and reference number - Jiang[32]

Line 476: after reference number should be “.” Instead of “,” and give a space between “.” and “If” (s [36],If)

Response: Thank you for your meticulous review. The formatting issues you identified, which were present in the first draft, have all been addressed. Furthermore, we have thoroughly reviewed the entire manuscript and corrected any grammatical and formatting errors to ensure it conforms to the journal's style guidelines.

Comment 2: Line 272 “Sample selection and data sources” the reasons to excluding the three types of companies as a sample should be explained.

Response: Thank you for your insightful comments. In response to your request for clarification on the exclusion criteria for certain company types, we have now detailed the reasons for excluding the following categories of companies in the revised manuscript:

Companies in special industries such as finance and insurance are excluded due to their distinct business models and risk profiles, which may compromise the generalizability of our findings.

Companies with incomplete data for key variables are excluded to avoid biases that could distort the research outcomes.

Companies with special designations like ST and *ST, as well as those with abnormal operations, are excluded because their financial data and stock price volatility are significantly different from those of companies operating under normal conditions.

We appreciate the opportunity to refine our manuscript based on your feedback and hope that our revisions meet with your approval.

Comment 3: Line 535: in the “Recommendation” section it would be better to include the research limitation and future research in relation to the ESG concept.

Response: Thank you for your valuable feedback. In response to your suggestion, we have incorporated a new section, "7.3 Research Limitations and Future Research Prospects," to address the limitations and potential avenues for future work.

We have acknowledged the lack of a unified ESG evaluation system and its implications for our study's findings. Additionally, we recognize the limitations in measuring investor attention using the Baidu index and suggest that more precise metrics are needed.

Looking ahead, we propose that future research should aim to develop a robust ESG rating framework and explore more accurate methods for gauging investor attention. We also recommend examining the impact of ESG performance on stock price volatility across various industries and regions, as well as the influence of individual ESG components.

We trust that these revisions provide a clearer understanding of our study's scope and limitations, and we appreciate the opportunity to enhance our manuscript based on your guidance.

**Response to the comments of**

**Reviewer #7**

 Comment 1：The context of research is ommited in the introduction section. The motivation and the contribution of the study are presented, but the readers can not find that they were be supported by privious studies.

Response: Thank you for your insightful feedback. We have revised the introduction to include a theoretical framework and a concise literature review that support our research context and contributions.

In the second paragraph, we've incorporated risk management theories to elucidate the risk reduction effects of ESG performance across subcomponents. We've also provided a brief review of the literature on ESG's impact on abnormal stock price volatility, noting the existing lack of consensus.

The third paragraph now includes insights from signaling and reputation theories, underscoring the existing literature's findings on the link between ESG performance and investor attention, and the potential mediating role of investor attention in the relationship between ESG performance and stock price volatility. We've identified a gap in the literature regarding this mediating mechanism.

The revised introduction is now grounded in theory and supported by literature. We hope these revisions meet your expectations.

Comment 2：Theorization is average, the literature review section were presented by listing. The backgroud literature is missing. The readers will question whether the authors are based on what literature or theories to propose variables as Equation 1,2,3. While carrying out the review of literature, the authors should break the review into different themes, based on their choice of variables. It will not only make the review of literature more systematic, but also will provide a rationale behind choice of the variables. Moreover, the literature review should provide a basis for the study by substantiating the research gap.

Response: Thank you for your insightful suggestions. We have thoroughly revised the literature review section, organizing it thematically around our research hypotheses and variable selection to provide a clear theoretical basis for our study.

We have removed the original literature review on the economic consequences of ESG performance, as it did not directly relate to our research hypotheses. Instead, we have focused the review on the relationship between a firm's ESG performance and abnormal stock price volatility, as well as the impact of investor concerns on stock price volatility. This thematic approach not only systematizes the literature review but also directly supports the rationale behind our variable selection.

Our review highlights the lack of consensus in the existing literature on the effects of ESG performance on stock price volatility and identifies a significant gap regarding the mediating role of investor attention. This study aims to address these gaps, and the revised literature review now serves as a robust foundation for our research, substantiating the research gap and supporting our theoretical framework.

We trust that these enhancements meet your expectations and provide a solid basis for our study.

Comment 3：The authors used several approaches. However, please discuss why them is adopted? What are the conditions and advantages of them?

Response: Thank you for your constructive suggestions. We have carefully considered your feedback regarding the use of various methods for robustness and endogeneity tests in our study. In the revised manuscript, we have provided a detailed discussion on the rationale behind the selection of each method, along with their respective conditions and advantages. Specifically, we have:

Addressed the lack of standardized ESG ratings by replacing the explanatory variables to ensure the robustness of our results. This approach acknowledges the variability in evaluation criteria and coverage among rating agencies.

Employed the Generalized Method of Moments (GMM) estimation to verify the robustness of our dynamic panel data model, which is particularly advantageous for dealing with potential issues of endogeneity inherent in such models.

Utilized Propensity Score Matching (PSM) to mitigate endogeneity arising from sample selection bias, recognizing that a firm's ESG performance may be influenced by its financial status and governance.

When we use each method in the manuscript, we briefly describe the reasons, conditions, and advantages of using that method.We trust that these revisions provide a comprehensive understanding of our methodological choices and their relevance to our study.

Comment 4：The Pooled OLS and GMM approaches have some limitations. In the “Empirical results” section, please provide more diagnostic tests, such as slope heterogeneity test. I think there might be a problem with slope heterogeneity among the sampled countries. Please show advantages of these approaches for the readers.

Response: Thank you for your meticulous review and valuable suggestions. Your comments on the limitations of the Pooled OLS and GMM methods are very pertinent and are essential to improve the quality and credibility of our study.

In response to your proposal for a slope heterogeneity test, we have given it in-depth consideration and discussion. We recognize that the slope heterogeneity test is an important tool for diagnosing potential nonlinearities in the model or differences between different subsamples. However, in the current study, given the large differences in economic structures and policy environments between our samples, we believe that the slope heterogeneity test may not adequately capture these complex dynamics. Therefore, we opted for a diagnostic test that is more appropriate to the context of our study.

To enhance the robustness of our results, we add the following diagnostic tests in the "Empirical results" section:(1) Propensity Score Matching (PSM): We used PSM to address the issue of potential selection bias by ensuring that the treatment and control groups were similar on observable characteristics prior to treatment. (2) Instrumental variables approach: we chose appropriate instrumental variables for two-stage least squares regression analyses to address possible endogeneity issues.

In addition, we agree that the reader needs to be aware of the advantages of the methods we employ. In the revised manuscript, we describe the advantages of these methods.By adding more diagnostic tests and elaborating on the advantages of these methods, our study is more comprehensive and reliable.

Comment 5：The policy recommendations are weak and sound more generic. Authors need to provide policy recommendations in accordance with the results. Can you please be direct about what policies that administrators in China should be implemented? And, why?

Response: Thank you for your insightful comments. We have revised our policy recommendations to include three actionable measures for businesses, investors and government agencies. In particular, the recommendations for government agencies detail the specific policies that should be implemented and why. This includes three main points:

(1) Provide ESG financial incentives: We recommend that the government provide subsidies and tax incentives to encourage listed companies to enhance their environmental and social responsibilities and improve the ESG disclosure system. (2) Enhanced environmental regulation: We suggest stricter pollution control through an emissions licensing system, tougher penalties for "greenwashing", and incentives for compliance through market mechanisms such as emissions charges. (3) Enhance ESG Information Transparency: We recommend mandatory and free public disclosure of ESG ratings to ensure that investors have access to accurate information, reduce stock price volatility, and support capital market stability.

These recommendations are based on the results of our empirical research, which emphasizes the significant impact of corporate ESG performance on stock price volatility and investor decision-making. We have ensured that the revised manuscript clearly articulates the rationale behind each of the policy recommendations and how they can be effectively implemented.

We believe that these revisions have greatly improved the quality of the manuscript. We are grateful for your professional comments, which have pointed out the issues. We hope that our revisions meet your expectations.

Thank you for your time and consideration.

Sincerely,

Fengju Wu

E-mail: wufengju@naujsc.edu.cn
